# The length of proximal margin does not influence the prognosis of Siewert type II/III adenocarcinoma of esophagogastric junction after transhiatal curative gastrectomy

**DOI:** 10.1186/s40064-016-2240-3

**Published:** 2016-05-11

**Authors:** Fan Feng, Yangzi Tian, Guanghui Xu, Shushang Liu, Zhen Liu, Gaozan Zheng, Man Guo, Xiao Lian, Daiming Fan, Hongwei Zhang

**Affiliations:** Department of Digestive Surgery, Xijing Hospital, Fourth Military Medical University, 127 West Changle Road, Xi’an, 710032 Shaanxi China; Department of Dermatology, Xijing Hospital, Fourth Military Medical University, 127 West Changle Road, Xi’an, 710032 Shaanxi China

**Keywords:** Proximal margin, Siewert type, Adenocarcinoma of esophagogastric junction, Prognosis

## Abstract

**Background:**

The optimal length of proximal margin for Siewert type II/III adenocarcinoma of the esophagogastric junction (AEJ) is still need to be clarified. The aim of the present study was to investigate the appropriate length of proximal margin for Siewert type II/III AEJ through transhiatal approach.

**Methods:**

From September 2009 to December 2014, a total of 693 consecutive patients with Siewert type II/III AEJ were retrospectively analyzed. All patients received transhiatal R0 resection. The proximal margin length was measured immediately after resection. The prognostic value of proximal margin length on Siewert type II/III AEJ with transhiatal approach was analyzed.

**Results:**

There were 404 cases of Siewert type II AEJ (58.3 %) and 289 cases of Siewert type III AEJ (41.7 %). Total gastrectomy was performed in 526 patients (75.9 %), and proximal gastrectomy was performed in 167 patients (24.1 %). The median length of the gross proximal margin was 2.4 (range 0.1–5.0) cm. Lymph node metastasis was the only independent prognostic predictor for Siewert type II AEJ. Tumor size and lymph node metastasis were independent prognostic predictors for Siewert type III AEJ.

**Conclusions:**

For Siewert type II/III AEJ with esophageal invasion of 3 cm or less, proximal margin length does not influence the prognosis of patients after transhiatal curative gastrectomy.

## Background

Adenocarcinoma of the esophagogastric junction (AEJ) is defined as a tumor with an epicenter within the 5 cm proximal and distal of the esophagogastric junction (Keeney and Bauer 2006). The incidence of AEJ has been increasing in both Western and Asian countries (Hasegawa and Yoshikawa 2010). AEJ is classified into three types by Siewert in 1998 (Siewert and Stein 1998). Type I is defined as tumors with an epicenter of 1–5 cm above the junction, type II as epicenter within 1 cm above and 2 cm below the junction, type III as epicenter within 2–5 cm below the junction (Parry et al. 2015). There are many unresolved issues on surgical management. Curative surgical resection is considered the mainstay of therapy. The current trend of surgical resection for Siewert type III AEJ was radical gastrectomy (Gertler et al. 2011). However, the optimal surgical treatment for Siewert type II AEJ remains controversial. Previously, two phase III clinical trials performed in Japan and Netherlands demonstrated that transthoracic approach could not improve the prognosis of patients with Siewert type II compared with transhiatal approach (Sasako et al. 2006; Hulscher et al. 2002). Thus, transhiatal approach is considered sufficient for Siewert type II AEJ.

The optimal length of proximal margin for Siewert type II/III AEJ is still need to be clarified. Several reports have advocated that a resection margin of up to 10 cm is necessary to prevent local recurrence (Ito et al. 2004; Mariette et al. 2003). Barbour et al. reported that the prognosis of T2+ patients could be significantly improved if the length of proximal margin was greater than 3.8 cm (Barbour et al. 2007). A recent study revealed that proximal margin length of more than 2 cm seemed satisfactory for patients with type II/III AEJ (Mine et al. 2013). Thus, the aim of the present study was to investigate the appropriate length of proximal margin for Siewert type II/III AEJ through transhiatal approach.

## Patients and methods

This study was performed in the Xijing Hospital of Digestive Diseases affiliated to the Fourth Military Medical University. From September 2009 to December 2014, a total of 693 consecutive patients with AEJ were retrospectively analyzed. The inclusion criteria were: (1) Siewert type II or III AEJ, (2) length of esophageal invasion was less than 3 cm, (3) underwent radical proximal or total gastrectomy, (4) with negative proximal margin, (5) pathological T2-4N0-3M0 tumor. The exclusion criteria were: (1) with neoadjuvant therapy, (2) with malignant tumor in other location, (3) underwent left thoracoabdominal approach. This study was approved by the Ethics Committee of Xijing Hospital, and written informed consent was obtained from all patients before surgery.

All patients were treated with proximal or total gastrectomy with a combined D2 lymphadenectomy via a transhiatal approach. The proximal margin was sent for frozen-section pathological examination when the proximal margin length was considered inadequate. If the proximal margin was positive, further resection of distal esophagus with an additional 1–2 cm was performed. All the surgical procedure and the extent of lymph node clearance were based on the recommendations of the Japanese Gastric Cancer Treatment Guidelines (Japanese Gastric Cancer Association 2011).

The fresh specimen was cut open longitudinally immediately after resection. Then the specimen was stretched maximally and fixed on a board. The length of proximal margin was measured and recorded by the surgeon. Additional proximal margin length was also measured if further resection of distal esophagus was performed. Then, specimens were fixed in 10 % neutral formalin immediately and embedded routinely for pathological examination.

Clinicopathological features including age, gender, tumor size, differentiation status, Bormann type, length of proximal margin, tumor depth, lymph node metastasis, lymphatic–vascular invasion (LVI), neural invasion, operation time, blood loss and postoperative complications were collected. The patients were followed up till October 2015 by enhanced chest and abdominal CT every 6 months after discharge to evaluate tumor recurrence and distant metastasis.

Data were processed using SPSS 22.0 for Windows (SPSS Inc., Chicago, IL, USA). Discrete variables were analyzed using the Chi square test or Fisher’s exact test. Numerical variables were expressed as the mean ± SD unless otherwise stated. Significant predictors for survival identified by univariate analysis were further assessed by multivariate analysis using the logistic regression analysis. The P value was considered to be statistically significant at 5 % level.

## Results

The clinicopathological and surgical related characteristics of patients were summarized in Table 1. There were 603 male (87.0 %) and 90 female (13.0 %). The patient age ranged from 22 to 87 years (median 61 years; mean 60.4 years). There were 404 cases of Siewert type II AEJ (58.3 %) and 289 cases of Siewert type III AEJ (41.7 %). Total gastrectomy was performed in 526 patients (75.9 %), and proximal gastrectomy was performed in 167 patients (24.1 %). The median length of the gross proximal margin was 2.4 (range 0.1–5.0) cm. The 5-year overall survival of Siewert type II and III was 54.7 and 50.8 %, respectively.

**Table 1:** Clinicopathological and surgical related characteristics of gastric cancer patients

Characteristics	Parameters
Age	
≤60	330 (47.6 %)
>60	363 (52.4 %)
Gender	
Male	603 (87.0 %)
Female	90 (13.0 %)
Siewert type	
II	404 (58.3 %)
III	289 (41.7 %)
Type of resection	
Proximal gastrectomy	167 (24.1 %)
Total gastrectomy	526 (75.9 %)
Length of proximal margin	2.24 ± 0.99 cm
Tumor size	
≤5 cm	410 (59.2 %)
>5 cm	283 (40.8 %)
Differentiation status	
Well	54 (7.8 %)
Moderately	254 (36.7 %)
Poor	348 (50.2 %)
Signet ring cell or mucinous	37 (5.3 %)
Bormann type	
I	81 (11.7 %)
II	161 (23.2 %)
III	373 (53.8 %)
IV	78 (11.3 %)
Tumor depth	
T2	92 (13.3 %)
T3	353 (50.9 %)
T4	248 (35.8 %)
Lymph node metastasis	
N0	189 (27.3 %)
N1	154 (22.2 %)
N2	143 (20.6 %)
N3	207 (29.9 %)
TNM stage	
I	54 (7.8 %)
II	259 (37.4 %)
III	380 (54.8 %)
Lymphatic–vascular invasion	
Yes	439 (63.3 %)
No	254 (36.7 %)
Neural invasion	
Yes	596 (86.0 %)
No	97 (14.0 %)
Operation time	218.62 ± 81.36 min
Intraoperative blood loss	243.72 ± 183.95 ml
Clavien–Dindo classification	
I	46 (51.1 %)
II	16 (17.8 %)
IIIA	5 (5.6 %)
IIIB	22 (24.4 %)
V	1 (1.1 %)

The prognostic factors for Siewert type II AEJ were summarized in Table 2. The results showed that tumor size, Borrmann type, tumor depth, lymph node metastasis, TNM stage, lymphatic–vascular invasion, neural invasion and intraoperative blood loss were risk factors for the prognosis of Siewert type II AEJ. However, only lymph node metastasis were independent prognostic predictors (Table 3). The prognostic factors for Siewert type III AEJ were summarized in Table 4. The results showed that tumor size, lymph node metastasis, TNM stage, lymphatic–vascular invasion, neural invasion and intraoperative blood loss were risk factors for the prognosis of Siewert type II AEJ. However, only tumor size and lymph node metastasis were independent prognostic predictors (Table 5). It was worth mentioning that the proximal margin length was not a prognostic predictor for Siewert type II/III AEJ.

**Table 2:** Univariate analysis of prognostic factors for Siewert type II patients

Prognostic factors	β	Hazard ratio (95 % CI)	*P* value
Age	0.354	1.425 (0.990–2.052)	0.057
Gender	0.169	1.184 (0.718–1.954)	0.508
Type of resection	0.366	1.443 (0.979–2.124)	0.064
Length of proximal margin	0.063	1.066 (0.885–1.282)	0.502
Tumor size	0.549	1.732 (1.203–2.494)	0.003
Differentiation status	0.034	1.035 (0.846–1.267)	0.739
Borrmann type	0.352	1.422 (1.104–1.831)	0.006
Tumor depth	0.578	1.782 (1.344–2.362)	0.000
Lymph node metastasis	0.532	1.702 (1.448–2.001)	0.000
TNM stage	0.945	2.573 (1.844–3.591)	0.000
Lymphatic–vascular invasion	1.089	2.970 (1.756–5.026)	0.000
Neural invasion	0.738	2.092 (1.040–4.209)	0.039
Operation time	0.002	1.002 (0.999–1.004)	0.145
Intraoperative blood loss	0.001	1.001 (1.000–1.002)	0.014
Postoperative morbidity (yes/no)	−0.083	0.921 (0.495–1.712)	0.794

**Table 3:** Multivariate analysis of prognostic factors for Siewert type II patients

Prognostic factors	β	Hazard ratio (95 % CI)	*P* value
Age	0.420	1.539 (0.907–2.611)	0.110
Gender	−0.103	0.902 (0.475–1.713)	0.753
Type of resection	0.319	1.376 (0.740–2.558)	0.314
Length of proximal margin	−0.035	0.965 (0.767–1.215)	0.764
Tumor size	−0.008	0.992 (0.594–1.656)	0.975
Differentiation status	0.219	1.245 (0.943–1.644)	0.122
Borrmann type	0.074	1.076 (0.758–1.530)	0.681
Tumor depth	0.111	1.117 (0.710–1.757)	0.632
Lymph node metastasis	0.693	2.000 (1.152–2.525)	0.000
TNM stage	0.169	1.184 (0.475–2.949)	0.717
Lymphatic–vascular invasion	0.523	1.688 (0.911–3.129)	0.096
Neural invasion	0.250	1.285 (0.604–2.731)	0.515
Operation time	0.002	1.002 (0.999–1.005)	0.269
Intraoperative blood loss	0.000	1.000 (0.999–1.001)	0.923
Postoperative morbidity (yes/no)	0.158	1.171 (0.551–2.488)	0.681

**Table 4:** Univariate analysis of prognostic factors for Siewert type III patients

Prognostic factors	β	Hazard ratio (95 % CI)	*P* value
Age	0.362	1.437 (0.961–2.149)	0.077
Gender	0.090	1.094 (0.598–2.002)	0.771
Type of resection	0.555	1.742 (0.873–3.477)	0.115
Length of proximal margin	−0.043	0.958 (0.783–1.171)	0.674
Tumor size	0.759	2.136 (1.397–3.266)	0.000
Differentiation status	−0.113	0.893 (0.683–1.167)	0.408
Borrmann type	0.097	1.102 (0.859–1.413)	0.444
Tumor depth	0.242	1.273 (0.921–1.762)	0.144
Lymph node metastasis	0.453	1.572 (1.305–1.894)	0.000
TNM stage	0.733	2.082 (1.407–3.082)	0.000
Lymphatic–vascular invasion	0.611	1.842 (1.066–3.184)	0.029
Neural invasion	1.027	2.792 (1.015–7.697)	0.047
Operation time	0.001	1.001 (1.000–1.003)	0.150
Intraoperative blood loss	0.001	1.001 (1.000–1.002)	0.014
Postoperative morbidity (yes/no)	0.185	1.203 (0.783–1.846)	0.399

**Table 5:** Multivariate analysis of prognostic factors for Siewert type III patients

Prognostic factors	β	Hazard ratio (95 % CI)	*P* value
Age	0.407	1.503 (0.898–2.513)	0.121
Gender	0.270	1.310 (0.651–2.637)	0.450
Type of resection	−0.415	0.661 (0.233–1.871)	0.435
Length of proximal margin	−0.030	0.970 (0.736–1.279)	0.831
Tumor size	0.555	1.742 (1.025–2.959)	0.040
Differentiation status	−0.021	0.979 (0.704–1.360)	0.899
Borrmann type	0.113	1.120 (0.817–1.535)	0.482
Tumor depth	0.190	1.210 (0.689–2.124)	0.508
Lymph node metastasis	0.401	1.493 (1.176–1.895)	0.001
TNM stage	−0.174	0.840 (0.303–2.334)	0.739
Lymphatic–vascular invasion	0.100	1.105 (0.600–2.035)	0.748
Neural invasion	0.479	1.615 (0.524–4.978)	0.404
Operation time	0.002	1.002 (0.998–1.006)	0.338
Intraoperative blood loss	0.000	1.000 (0.999–1.001)	0.964
Postoperative morbidity (yes/no)	0.060	1.061 (0.511–2.206)	0.873

## Discussion

The optimal length of proximal margin for Siewert type II/III AEJ remains controversial, especially for transhiatal approach. Thus, our present study mainly focused on the influence of proximal margin length on the prognosis of patients with Siewert type II/III AEJ via transhiatal approach. We found that the proximal margin length does not influence the prognosis of patients with Siewert type II/III AEJ.

The optimal surgical approach for Siewert type II AEJ has not yet been agreed upon, although both transthoracic and transhiatal approach for Siewert type II has been attempted in the past few years (Mariette et al. 2011). The transthoracic approach is based on the principle that the proximal margin length has a critical impact on the prognosis, and the transhiatal approach is based on evidence that the abdominal lymph node metastasis is common and also has a great impact on the survival (Yamashita et al. 2011). The main goal of either approach remains complete tumor remove. A Dutch group has performed a prospective randomized controlled trial to compare the right transthoracic and transhiatal approaches for Siewert I/II tumors. As no significant difference was noted for prognosis between the two approaches, transthoracic approach is not recommended for Siewert type II tumors (Hulscher et al. 2002). A Japanese group performed another prospective randomized controlled trial to compare the effect of left thoracoabdominal and transhiatal approaches for Siewert II/III tumors with esophageal invasion of 3 cm or less (Sasako et al. 2006). The result showed that left thoracoabdominal approach could not improve the survival and will result in increased morbidity after operation, although this approach enabled complete dissection of the lower mediastinal lymph nodes. A meta-analysis conducted by Wei et al. showed that there was no difference in overall survival for Siewert type II AEJ between transthoracic and transhiatal approach, but transhiatal approach could decrease pulmonary complications and hospital stay (Wei et al. 2014). Therefore, the transhiatal approach was recommended for the treatment of Siewert type II/III AEJ with esophageal invasion of 3 cm or less (Hosoda et al. 2015). It was reported that the length of esophageal invasion was associated with lower mediastinal lymph node metastasis (Nakamura et al. 2012). Thus, for Siewert type II tumors with esophageal invasion more than 3 cm, mediastinal lymph node dissection via a transthoracic approach may provide a therapeutic benefit (Kurokawa et al. 2015). Given this situation, only Siewert type II/III AEJ patients with esophageal invasion of 3 cm or less were enrolled in our present study.

Previously, only two studies investigate the impact of proximal margin length on the prognosis of AEJ. Barbour et al. demonstrated that proximal margin greater than 3.8 cm is associated with improved prognosis for patients with Siewert types I/II/III AEJ that have undergone R0 resection with more than 15 lymph nodes examined (Barbour et al. 2007). However, Mine et al. reported that gross proximal margin length of more than 2.0 cm seem satisfactory for patients with Siewert type II/III AEJ treated by transhiatal approach (Mine et al. 2013). In our present study, we found that the proximal margin length does not influence the survival of patients with Siewert type II/III AEJ with transhiatal approach and R0 resection. This indicated that negative proximal margin may be suficient during the surgical resection of Siewert type II/III tumors. The conflict result between our present study and previous reports may contribute to several reasons. The most likely reason was that the sample size in our present study was significant larger than that in the studies reported by Mine et al. (100 cases) and Barbour et al. (275 cases with more than 15 lymph nodes examined). Second, all the three studies were based on single center data, which will result in bias. Third, they were all retrospective analysis. Thus, multicenter perspective randomized controlled trial should be carried out.

In our present study, the incidence of positive proximal margin was approximately 2.0 % (data not shown). The incidence was similar to 3.0 % described by Barbour et al. (Barbour et al. 2007) and 1.4 % reported by Mine et al. (2013). It was reported that positive margin was associated with deeper invasion and tumor size (Shen et al. 2006). Patients with stage III and IV disease may have a higher risk of sub-clinical intramural tumor infiltration to the esophagus (Bozzetti et al. 2000). Thus, in high risk patients, intraoperative frozen section analysis is always used to evaluate the tumor involvement of the proximal margins.

The influence of positive margin on the survival of patients with AEJ remains controversial (Migliore et al. 2014). Marriette et al. have shown that the median survival time of patients with positive proximal margin was significantly lower than that with negative proximal margin (Mariette et al. 2003). However, DiMusto et al. found that 80 % of patients with positive proximal margin died with distant metastasis, which would not be influenced by more extensive resection (DiMusto and Orringer 2007). They also found that adjuvant therapy for a positive margin neither improves prognosis nor reduces local recurrence. Shen et al. also reported that positive margin was not an independent prognostic factor for survival (Shen et al. 2006). Thus, the necessity of extensive resection during operation when positive margin was demonstrated by frozen section evaluation and the necessity of reoperation when positive margin was found by routine pathological examination need further investigation.

There were several limitations in our present study. First, it was a retrospective analysis. To clarify the influence of proximal margin length on the prognosis of patients, a well-designed randomized clinical trial should be carried out. Second, the present analysis was limited to proximal margins ranging from 0 to 5.0 cm, the influence of proximal margins more than 5.0 cm were not evaluated. Third, the sample size was not large enough. This may result in bias during analysis. Fourth, the association between proximal margin length and anastomotic recurrence was not investigated. Fifth, the risk factors for positive proximal margin were not evaluated in our present study.

## Conclusions

For Siewert type II/III AEJ with esophageal invasion of 3 cm or less, proximal margin length does not influence the prognosis of patients after transhiatal curative gastrectomy.

## References

[CR1] Barbour AP, Rizk NP, Gonen M, Tang L, Bains MS, Rusch VW (2007). Adenocarcinoma of the gastroesophageal junction: influence of esophageal resection margin and operative approach on outcome. Ann Surg.

[CR2] Bozzetti F, Bignami P, Bertario L, Fissi S, Eboli M (2000). Surgical treatment of gastric cancer invading the oesophagus. Eur J Surg Oncol.

[CR3] DiMusto PD, Orringer MB (2007) Transhiatal esophagectomy for distal and cardia cancers: implications of a positive gastric margin. Ann Thorac Surg 831993–831998 **(discussion 1998–1999)** 10.1016/j.athoracsur.2006.09.025 17532385

[CR4] Gertler R, Stein HJ, Langer R, Nettelmann M, Schuster T, Hoefler H (2011). Long-term outcome of 2920 patients with cancers of the esophagus and esophagogastric junction: evaluation of the New Union Internationale Contre le Cancer/American Joint Cancer Committee staging system. Ann Surg.

[CR5] Hasegawa S, Yoshikawa T (2010). Adenocarcinoma of the esophagogastric junction: incidence, characteristics, and treatment strategies. Gastric Cancer.

[CR6] Hosoda K, Yamashita K, Katada N, Watanabe M (2015). Overview of multimodal therapy for adenocarcinoma of the esophagogastric junction. Gen Thorac Cardiovasc Surg.

[CR7] Hulscher JB, van Sandick JW, de Boer AG, Wijnhoven BP, Tijssen JG, Fockens P (2002). Extended transthoracic resection compared with limited transhiatal resection for adenocarcinoma of the esophagus. N Engl J Med.

[CR8] Ito H, Clancy TE, Osteen RT, Swanson RS, Bueno R, Sugarbaker DJ (2004). Adenocarcinoma of the gastric cardia: what is the optimal surgical approach?. J Am Coll Surg.

[CR9] Japanese Gastric Cancer Association (2011). Japanese gastric cancer treatment guidelines 2010 (ver. 3). Gastric Cancer.

[CR10] Keeney S, Bauer TL (2006). Epidemiology of adenocarcinoma of the esophagogastric junction. Surg Oncol Clin N Am.

[CR11] Kurokawa Y, Hiki N, Yoshikawa T, Kishi K, Ito Y, Ohi M (2015). Mediastinal lymph node metastasis and recurrence in adenocarcinoma of the esophagogastric junction. Surgery.

[CR12] Mariette C, Castel B, Balon JM, Van Seuningen I, Triboulet JP (2003). Extent of oesophageal resection for adenocarcinoma of the oesophagogastric junction. Eur J Surg Oncol.

[CR13] Mariette C, Piessen G, Briez N, Gronnier C, Triboulet JP (2011). Oesophagogastric junction adenocarcinoma: which therapeutic approach?. Lancet Oncol.

[CR14] Migliore M, Rassl D, Criscione A (2014). Longitudinal and circumferential resection margin in adenocarcinoma of distal esophagus and cardia. Future Oncol.

[CR15] Mine S, Sano T, Hiki N, Yamada K, Kosuga T, Nunobe S (2013). Proximal margin length with transhiatal gastrectomy for Siewert type II and III adenocarcinomas of the oesophagogastric junction. Br J Surg.

[CR16] Nakamura M, Iwahashi M, Nakamori M, Naka T, Ojima T, Iida T (2012). Lower mediastinal lymph node metastasis is an independent survival factor of Siewert type II and III adenocarcinomas in the gastroesophageal junction. Am Surg.

[CR17] Parry K, Haverkamp L, Bruijnen RC, Siersema PD, Ruurda JP, van Hillegersberg R (2015). Surgical treatment of adenocarcinomas of the gastro-esophageal junction. Ann Surg Oncol.

[CR18] Sasako M, Sano T, Yamamoto S, Sairenji M, Arai K, Kinoshita T (2006). Left thoracoabdominal approach versus abdominal-transhiatal approach for gastric cancer of the cardia or subcardia: a randomised controlled trial. Lancet Oncol.

[CR19] Shen JG, Cheong JH, Hyung WJ, Kim J, Choi SH, Noh SH (2006). Influence of a microscopic positive proximal margin in the treatment of gastric adenocarcinoma of the cardia. World J Gastroenterol.

[CR20] Siewert JR, Stein HJ (1998). Classification of adenocarcinoma of the oesophagogastric junction. Br J Surg.

[CR21] Wei MT, Zhang YC, Deng XB, Yang TH, He YZ, Wang ZQ (2014). Transthoracic vs transhiatal surgery for cancer of the esophagogastric junction: a meta-analysis. World J Gastroenterol.

[CR22] Yamashita H, Katai H, Morita S, Saka M, Taniguchi H, Fukagawa T (2011). Optimal extent of lymph node dissection for Siewert type II esophagogastric junction carcinoma. Ann Surg.

